# Comparison of the efficacy of fish oil and probiotic supplementation on glucose and lipid metabolism in patients with type 2 diabetes: a systematic review and network meta-analysis

**DOI:** 10.1186/s13098-024-01266-3

**Published:** 2024-01-22

**Authors:** Mei Zhang, Fan Yang, Qiu Feng, Yanghong Ou, Jiaxing Zhang, Haiyan Wan, Hongyi Cao, Peng Ning

**Affiliations:** Department of Endocrine and Metabolism, Chengdu Fifth People’s Hospital (The Second Clinical Medical College, Affiliated Fifth People’s Hospital of Chengdu University of Traditional Chinese Medicine), Geriatric Diseases Institute of Chengdu, Chengdu, China

**Keywords:** Fish oil, Probiotic, Type 2 diabetes, Glucose metabolism, Lipid metabolism, Network meta-analysis

## Abstract

**Background:**

Abnormalities in glucose and lipid metabolism contribute to the progression and exacerbation of type 2 diabetes mellitus (T2DM). Fish oil and probiotics are dietary supplements that have the potential to improve glucose and lipid metabolism. However, their efficacy remains unclear in T2DM patients.

**Methods:**

PubMed, Embase, and the Cochrane Library were retrieved to collect randomized controlled trials (RCTs) on the efficacy of fish oil or probiotic supplementation in T2DM patients from the database inception to December 13, 2023. Primary outcome indicators encompassed glycated hemoglobin (HbA1c), homeostatic model assessment for insulin resistance (HOMA-IR) and blood lipid profile (triglyceride (TG) and total cholesterol (TC). Secondary outcome indicators included inflammatory markers such as tumor necrosis factor -α (TNF-α) and adipocytokine (including leptin and adiponectin). The R software was used for statistical analysis, and GraphPad Prism was used for figure rendering.

**Results:**

A total of 60 RCTs involving 3845 T2DM patients were included in the analysis. The results showed that the probiotics (*Bifidobacterium, Lactobacillus, Lactococcus, Propionibacterium, *etc*.*) were more effective in reducing HOMA-IR than fish oil (Surca = 0.935). *Bifidobacterium* demonstrated the highest efficacy in reducing HbA1c levels (Surca = 0.963). Regarding lipid metabolism, fish oil was superior to probiotics in lowering TG and TC levels (Surca values of 0.978 and 0.902, respectively). Furthermore, fish oil outperformed probiotics in reducing TNF-α (Surca = 0.839) and leptin (Surca = 0.712), and increasing adiponectin levels (Surca = 0.742). Node-splitting analysis showed good consistency (P > 0.05 for direct, indirect, and network comparison across various interventions).

**Conclusions:**

In T2DM patients, fish oil was more effective than probiotics in regulating lipid metabolism. Probiotics outperformed fish oil in regulating glucose metabolism particularly; specifically, *Bifidobacterium* showed higher efficacy in reducing blood glucose.

**Supplementary Information:**

The online version contains supplementary material available at 10.1186/s13098-024-01266-3.

## Introduction

The global prevalence and mortality rate of type 2 diabetes mellitus (T2DM) have been increasing, partly attributed to high sugar and fat diets. T2DM, if poorly managed, often causes such complications as macrovascular, microvascular, and neuropathies [1]. Therefore, effective management to improve glucose and lipid metabolism is crucial for T2DM patients. Although drug therapies and lifestyle interventions (e.g., low carbohydrate diet [2]) are the main strategies to control T2DM, the demand for dietary supplements increases due to their beneficial effects on maintaining or improving metabolic functions, particularly in patients with diabetes mellitus [3]. Fish oil and probiotics are two major supplements that can improve conditions related to digestive system [4, 5], neurological diseases [6], and T2DM [7–11].

Despite growing interest in dietary supplements, the relative efficacy of fish oil and probiotic supplements on glucose and lipid metabolism remains elusive in people with T2DM. Therefore, this systematic review and network meta-analysis (NMA) aims to evaluate the relative efficacy of fish oil and probiotics in improving glucose and lipid metabolism in T2DM patients based on available randomized controlled trials (RCTs). The primary goals are to close current research gaps, offer more informative guidance for the clinical treatment of T2DM, and provide a scientific basis for the development of dietary supplement therapies for T2DM.

## Methods

This study was reported in accordance with the Preferred Reporting Items for Systematic Reviews and Meta-Analyses (PRISMA) statement for systematic Evaluation and Meta-Analysis [12]. The study protocol has been registered in the International Prospective Systems Evaluation Register (PROSPERO) (Registration no. CRD42023407998).

### Search strategies

PubMed, Embase, and Cochrane Library were retrieved to collect relevant RCTs. The search strategy was designed based on the combination of MeSH terms and free words. The detailed search strategy is shown in Additional file 1: Table S1. Additionally, the reference lists of previous systematic reviews and meta-analyses were searched to determine potentially eligible studies.

### Research selection

The inclusion criteria were designed in strict accordance with the PICOS principle: (1) Population: adult patients were clearly diagnosed with T2DM based on World Health Organization 1999 and American Diabetes Association criteria [13]. No restrictions were imposed on nationality or race; (2) Intervention: The experimental group received fish oil, docosahexaenoic acid (DHA), eicosapentaenoic acid (EPA), one or more probiotics; (3) Comparison: The control group received vegetable oil, mineral oil, or placebo, regardless of doses and course of administration. If a study involved different study duration periods, the longest one was taken as the standard; if different doses were used, the largest one was taken as the standard; (4) Outcome: Studies had to report at least of the following outcome indicators: glycated hemoglobin (HbA1c), homeostatic model assessment for insulin resistance (HOMA-IR), triglyceride (TG), total cholesterol (TC), tumor necrosis factor-α (TNF-α), leptin, adiponectin; (5) Study design: The studies had to be RCTs. The following studies were excluded: (1) review, systematic evaluation, abstract, conference, retrospective study, cross-sectional study; (2) Studies that reported no relevant outcome indicators or no data could be extracted; (3) animal and cell tests; (4) Studies on patients with gestational diabetes; (5) non-English studies.

Two researchers (QF and YO) independently screened the studies. The titles and abstracts were checked to select potentially eligible articles. Then, a full-text review was conducted to identify eligible articles. Any dissents were resolved by a third researcher (PN).

### Data extraction

Data were independently extracted by two researchers (MZ and FY) using a predesigned spreadsheet. The extracted data included author, publication year, country, sample size, mean age, comparison and treatment details (fish oil and probiotics type, placebo), outcome indicators (HbA1c, HOMA-IR, TG, TC, TNF-α, leptin, adiponectin). Any inconsistencies in their results were adjudicated by a third researcher (PN).

### Risk-of-bias assessment

Two researchers (HW and HC) independently employed the Cochrane Risk Bias Tool version 2.0 to evaluate the risk of bias in the included studies, involving random sequence generation (selection bias), allocation concealment (selection bias), blinding of participants and personnel (performance bias), blinding of outcome assessment (detection bias), incomplete outcome data (attrition bias), selective reporting (reporting bias), and other bias. The study quality was rated as low risk of bias, some concerns, and high risk of bias. Any dissents between the two researchers were resolved by a third researcher (JZ).

### Data analysis

R version 4.3.1 (R Core Team, Vienna, Austria) was used for statistical analysis, and GraphPad Prism version 9.4.1 (GraphPad Software, San Diego, USA) was used for figure plotting. A network diagram was drawn to show all the available evidence for each intervention. The heterogeneity was determined by the I^2^ statistic. An I^2^ ≤ 50% indicated small or no heterogeneity between studies, and the fixed-effects model was employed; otherwise, the random-effects model was adopted [14]. The number of tuning and simulation iterations was set at 5000 and 20,000, respectively. The results were presented as mean difference (MD) with 95% confidence intervals (CIs), and the data were not statistically significant when the 95% CI value contained 0. The surface under the cumulative ranking curve (SUCRA) was used to calculate the probability of each intervention becoming the best intervention. The SUCRA value ranges from 0 to 1. A higher SUCRA value indicated a greater possibility of a treatment method becoming the most effective intervention [15]. To establish a network closed-loop structure, a node-splitting analysis was employed to evaluate the consistency of direct, indirect, and network comparisons across various interventions. The included studies were categorized into two groups based on intervention duration: < 12 weeks and ≥ 12 weeks. Then, network meta-analysis was performed within the groups. A value of α = 0. 05 was considered statistically significant.

## Results

### Research selection and characteristics

**Fig. 1:**
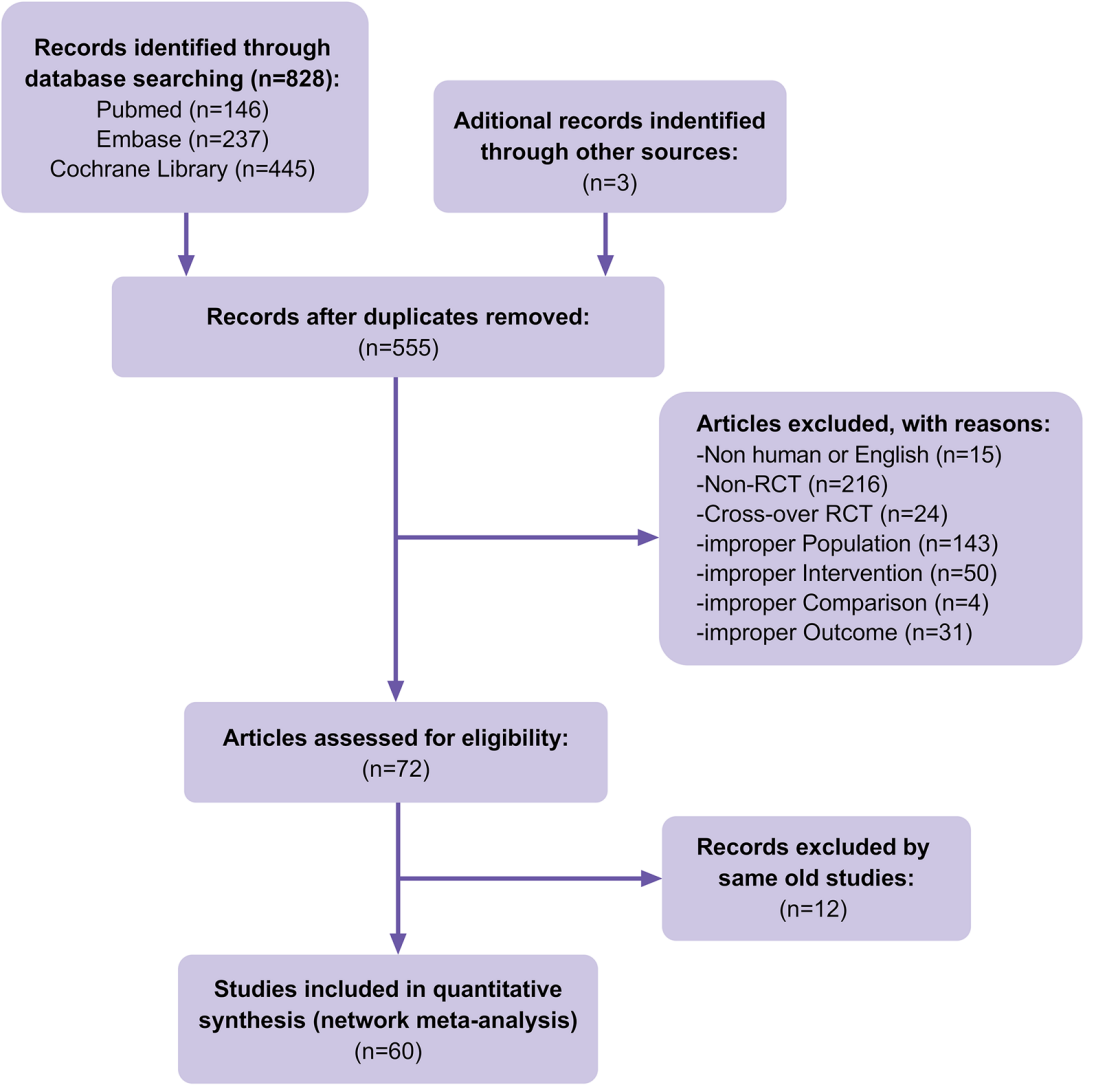
Flow of trials through the review

A total of 828 articles were identified in the initial database search from the database inception to December 13, 2023. Besides, the reference lists of previous systematic reviews and meta-analyses were also manually searched, and additional 3 eligible articles were found. Furthermore, 555 papers were left after eliminating duplicate papers. Then, we excluded 15 nonhuman studies or non-English studies, 240 non-RCT or cross-RCT studies, 228 articles that did not conform to the PICO principles of the study, and 12 duplicate publications. Finally, 60 RCTs (Fig. 1) involving 3845 T2DM patients were included in the analysis. The baseline characteristics of the included studies are shown in Tables 1 and 2.

**Table 1:** Characteristics of RCTs about the efficacy of fish oil in patients with type 2 diabetes

Study	Region	Intervention			Comparison			Follow-up	Outcome
		N	Age	Method	N	Age	Method		
Morgan [16]	USA	20	54.3 ± 7.5	Fish oil	20	54.9 ± 6.5	Corn oil	12 weeks	2–4
Sirtori [17]	Italy	203	NA	Fish oil	211	NA	Olive oil	12 months	2
Patti [18]	Italy	8	56.0 ± 8.5	Fish oil	8	57.0 ± 5.7	Olive oil	6 months	2–4
Woodman [19]	Australia	17/18	61.2 ± 9.6/60.9 ± 8.2	EPA/DHA	16	61.5 ± 7.6	Olive oil	6 weeks	2–4
Pedersen [20]	Denmark	23	NA	Fish oil	21	NA	Corn oil	8 weeks	2–4
Mita [21]	Japan	30	59.0 ± 11.2	EPA	30	61.2 ± 8.4	Placebo	2.1 years	2–4
Satoh [22]	Japan	22	51.6 ± 13.1	EPA	22	51.6 ± 15.0	Placebo	3 months	2–4
Kabir [23]	France	12	55.0 ± 6.9	Fish oil	14	55.0 ± 3.7	Paraffin oil	2 months	2–7
Shidfar [24]	Iran	25	53.4 ± 11.7	Fish oil	25	54.1 ± 11.1	Placebo	10 weeks	2–4
Wong [25]	China	49	61.2 ± 9.0	Fish oil	48	59.0 ± 9.3	Olive oil	12 weeks	3,4
Moghadam [26]	Iran	42	55.4 ± 9.9	Fish oil	42	53.0 ± 10.7	Sunflower oil	8 weeks	5
Crochemore [27]	Brazil	14	60.6 ± 7.4	Fish oil	13	61.8 ± 9.1	Placebo	1 month	1–4
Ogawa [28]	Japan	13	79.5 ± 8.6	Fish oil	13	81.2 ± 7.6	Placebo	3 months	2–5,7
Sarbolouki [29]	Iran	32	45.0 ± 4.9	EPA	35	45.3 ± 3.9	Corn oil	12 weeks	1,2
Toupchian [30]	Iran	35	55.8 ± 7.6	Fish oil	33	56.0 ± 7.0	Paraffin oil	8 weeks	1,3,4
Zheng [31]	China	63	59.7 ± 8.8	Fish oil	122	59.4 ± 10.5	Flaxseed and Corn oil	6 months	1–4
Mazaherioun [32]	Iran	44	51.2 ± 7.5	Fish oil	41	50.6 ± 7.2	Paraffin oil	10 weeks	3,4
Mazaherioun [33]									1,2,7
Jacobo-Cejudo [34]	Mexico	29	NA	Fish oil	25	NA	Placebo	24 weeks	1–4,6,7
Wang [35]	China	49	64.6 ± 5.5	Fish oil	50	66.3 ± 5.1	Corn oil	6 months	2–4
Fayh [36]	Brazil	15	50.5 ± 6.1	Fish oil	15	50.7 ± 6.7	Placebo	8 weeks	2–4
Raygan [37]	Iran	30	64.1 ± 9.3	Fish oil	30/30	64.6 ± 9.1/62.0 ± 13.0	Flaxseed oil/Paraffin oil	12 weeks	1,3,4
Rampally [38]	India	14	NA	Fish oil	14	NA	Placebo	3 months	2
Thota [39]	Australia	17	58.0 ± 2.5	Fish oil	16	50.0 ± 2.5	Corn oil	12 weeks	2
Golzari [40]	Iran	18	44.4 ± 3.8	EPA	18	44.7 ± 4.7	Placebo	8 weeks	2–4
Hua [41]	China	51	45.6 ± 5.9	Fish oil	54	43.7 ± 8.6	Corn oil	3 months	1–4,7
Naeini [42]	Iran	25	54.7 ± 7.6	Fish oil	25	56.3 ± 7.8	Paraffin oil	8 weeks	3,4
Golpour [43]	Iran	31	51.2 ± 7.5	Fish oil	30	50.6 ± 7.2	Placebo	10 weeks	1,2
Liu [44]	China	52	62.3 ± 7.6	Fish oil	50	63.8 ± 9.7	Perilla oil	6 months	2–4
Kuang [45]	China	44	59.8 ± 9.9	Fish oil	45	62.2 ± 9.4	Corn oil	2 months	3,4

1.HOMA-IR; 2.HbA1c; 3.TG; 4.TC; 5.TNF-α; 6.Leptin; 7.Adiponectin

**Table 2:** Characteristics of RCTs about the efficacy of probiotics in patients with type 2 diabetes

Study	Region	Intervention			Comparison			follow-up	Outcome
		N	Age	Method	N	Age	Method		
Ejtahed [46]	Iran	30	50.9 ± 1.4	B.,L.,S.	30	51.0 ± 1.3	L., S.	6 weeks	3,4
Ejtahed [47]									2
Asemi [48]	Iran	27	50.5 ± 9.8	B.,L.,S.	27	52.6 ± 7.1	Placebo	8 weeks	1–4
Tajadadi-Ebrahimi [49]	Iran	27	52.0 ± 7.2	L.	27	53.4 ± 7.5	Placebo	8 weeks	1
Shakeri [50]									4
Mohamadshahi [51]	Iran	21	53.0 ± 5.9	B.,L.,S.	21	49.0 ± 7.1	L., S.	8 weeks	2,5
Mohamadshahi [52]									3,4
Ostadrahimi [53]	Iran	30	NA	B.,L.,S.	30	NA	L., S.	8 weeks	2–4
Feizollahzadeh [54]	Iran	20	56.9 ± 8.1	L.	20	53.6 ± 7.2	Placebo	8 weeks	3,5,7
Rezaei [55]	Iran	45	50.5 ± 10.9	B.,L.	45	50.1 ± 9.2	L., S.	4 weeks	2–4
Tonucci [56]	Brazil	23	51.8 ± 6.6	B.,L.	22	51.0 ± 7.2	S.	6 weeks	4
Firouzi [57]	Malaysia	68	52.9 ± 9.2	B.,L.	68	54.2 ± 8.3	Placebo	12 weeks	1–4
Sabico [58]	UK	39	48.0 ± 8.3	B., L., La.	39	46.6 ± 5.9	Placebo	12 weeks	3,4
Sato [59]	Japan	34	64.0 ± 9.2	L.	34	65.0 ± 8.3	Placebo	16 weeks	2–5,7
Mobini [60]	Sweden	14	64.0 ± 6.0	L.	15	65.0 ± 5.0	Placebo	12 weeks	2–4,6,7
Abbasi [61]	Iran	20	56.9 ± 8.1	L.	20	53.6 ± 7.2	Placebo	8 weeks	3,4
Kobyliak [62]	Ukraine	31	52.2 ± 9.7	B., L., La., P.	22	57.2 ± 9.7	Placebo	8 weeks	1
Hsieh [63]	China	46	NA	L.	22	NA	Placebo	6 months	1–5
Raygan [64]	Iran	30	60.7 ± 9.4	B.,L.	30	61.8 ± 9.8	Placebo	12 weeks	1,3,4
Madempudi [65]	India	37	NA	B.,L.	37	NA	Placebo	12 weeks	1–4
Lestari [66]	Indonesia	16	NA	B.,L.	16	NA	L.,S.	4 weeks	4
Razmpoosh [67]	Iran	30	58.6 ± 6.5	B.,L.,S.	30	61.3 ± 5.2	Placebo	6 weeks	1,3,4
Khalili [68]	Iran	20	44.0 ± 8.1	L.	20	45.0 ± 5.37	Placebo	8 weeks	1,2
Palacios [69]	Australia	30	61.4 ± 8.9	B.,L.,S.	30	56.1 ± 12.3	Placebo	12 weeks	1,2
Perraudeau [70]	USA	42	51.5 ± 12.7	B.	16	53.5 ± 8.0	Placebo	12 weeks	1,2
Jiang [71]	China	42	56.0 ± 8.5	B.,L.,S.	34	56.1 ± 8.2	Placebo	12 weeks	2
Mirjalili [72]	Iran	36	54.5 ± 8.0	B.,L.	36	58.1 ± 9.8	Placebo	12 weeks	2–4
Chaiyasut[73]	Thailand	20	63.9 ± 1.4	B.	20	61.1 ± 1.8	Placebo	12 weeks	2–4
Savytska [74]	Ukraine	34	53.8 ± 9.6	B.L.La.P.	34	56.9 ± 9.9	Placebo	8 weeks	2
Zikou [75]	Greece	46	64.5 ± 11.1	B.,L.	45	65.7 ± 10.8	Placebo	6 months	2–4

1. HOMA-IR; 2.HbA1c; 3.TG; 4.TC; 5.TNF-α; 6.Leptin; 7.Adiponectin

*B*. *Bifidobacterium*, *L*. *Lactobacillus*, *La*. *Lactococcus*, *S*. *Streptococcus*, *P*. Propionibacterium

### Risk of bias in studies

**Fig. 2:**
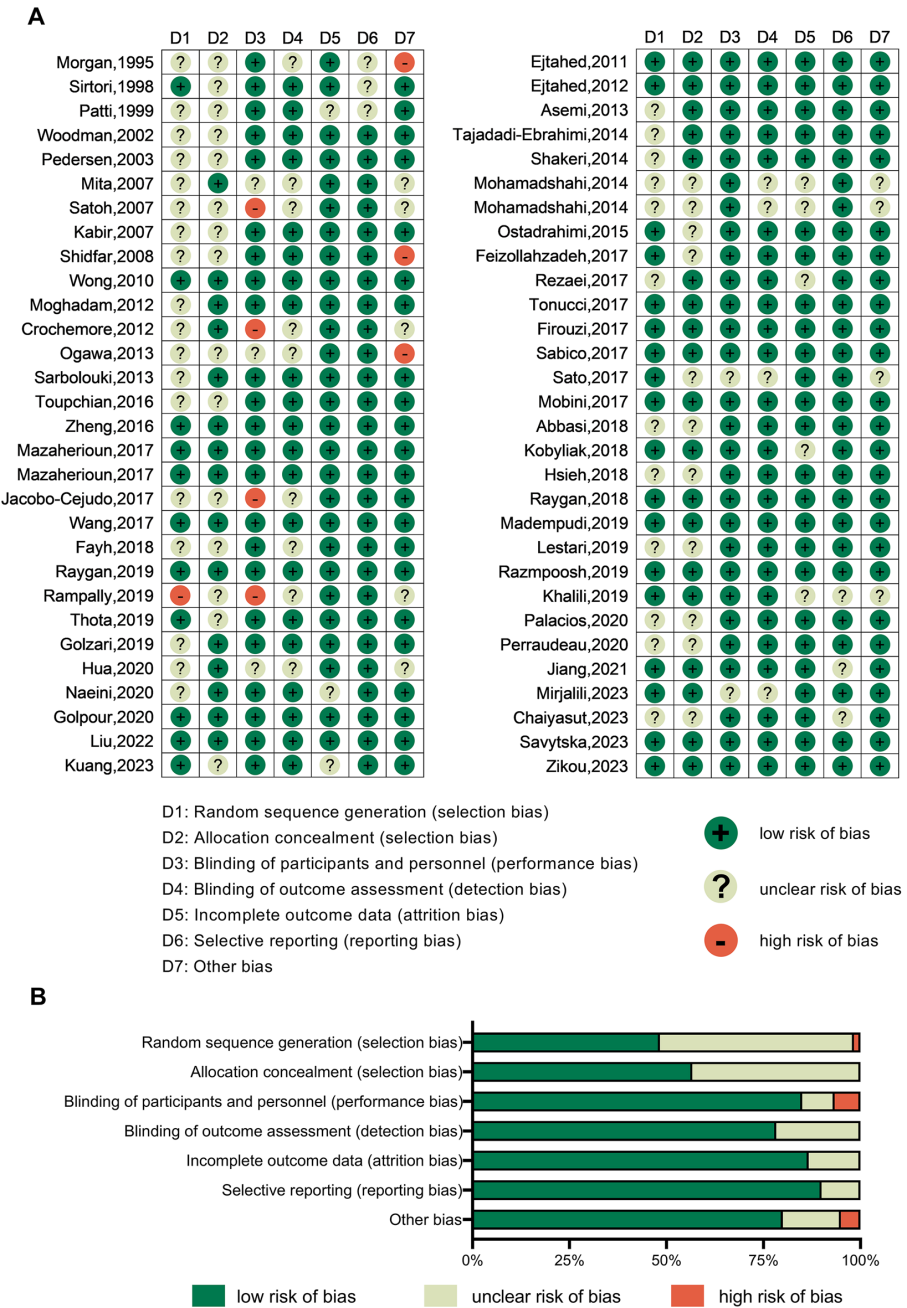
Risk-of-bias graph. **A** Risk-of-bias summary: review authors’ judgments about each risk-of-bias item for each included study. **B** Risk-of-bias graph: judgments about each risk-of-bias item presented as percentages across all included studies

The risk of bias in the included RCTs was assessed. Among the 60 included studies, 31 studies did not specifically describe the generation of random sequences; 26 studies did not specifically describe the allocation concealment; 9 did not specifically describe the method of blinding participants and implementers; 13 did not specifically describe the blinding of the outcome measurement; 8 studies contained incomplete data; 6 studies selectively reported their results; and 12 studies possessed other biases. The risk of bias in the included studies is illustrated in Fig. 2.

### Glucose metabolism

**Fig. 3:**
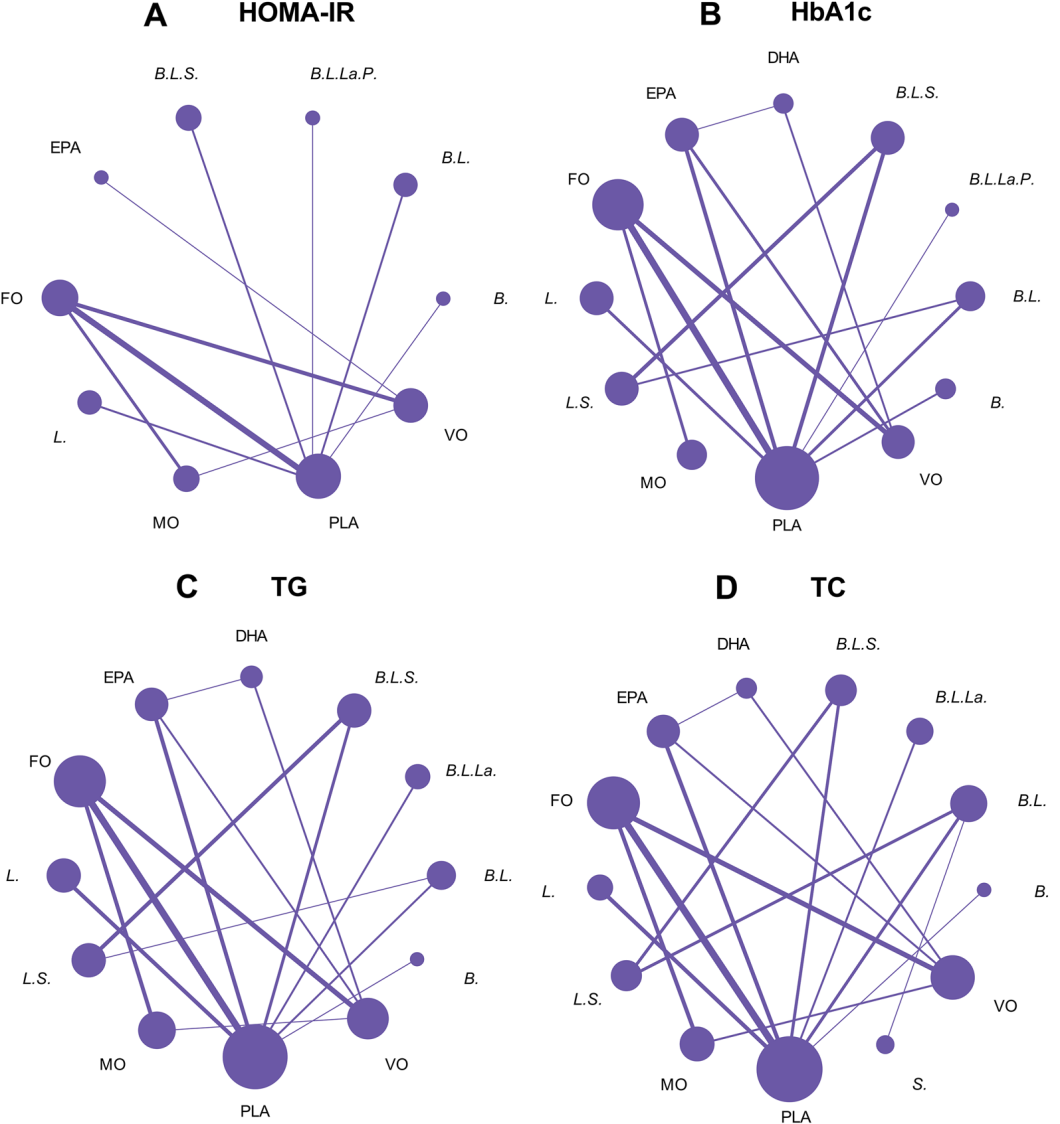
Network plots. *FO* Fish oil, *MO* Mineral oil including paraffin oil, *VO* Vegetale oil including corn oil, olive oil, sunflower oil, flaxseed oil and perilla oil, *B. Bifidobacterium, L. Lactobacillus, La. Lactococcus, S. Streptococcus, P. Propionibacterium*; *PLA* Placebo

**Fig. 4:**
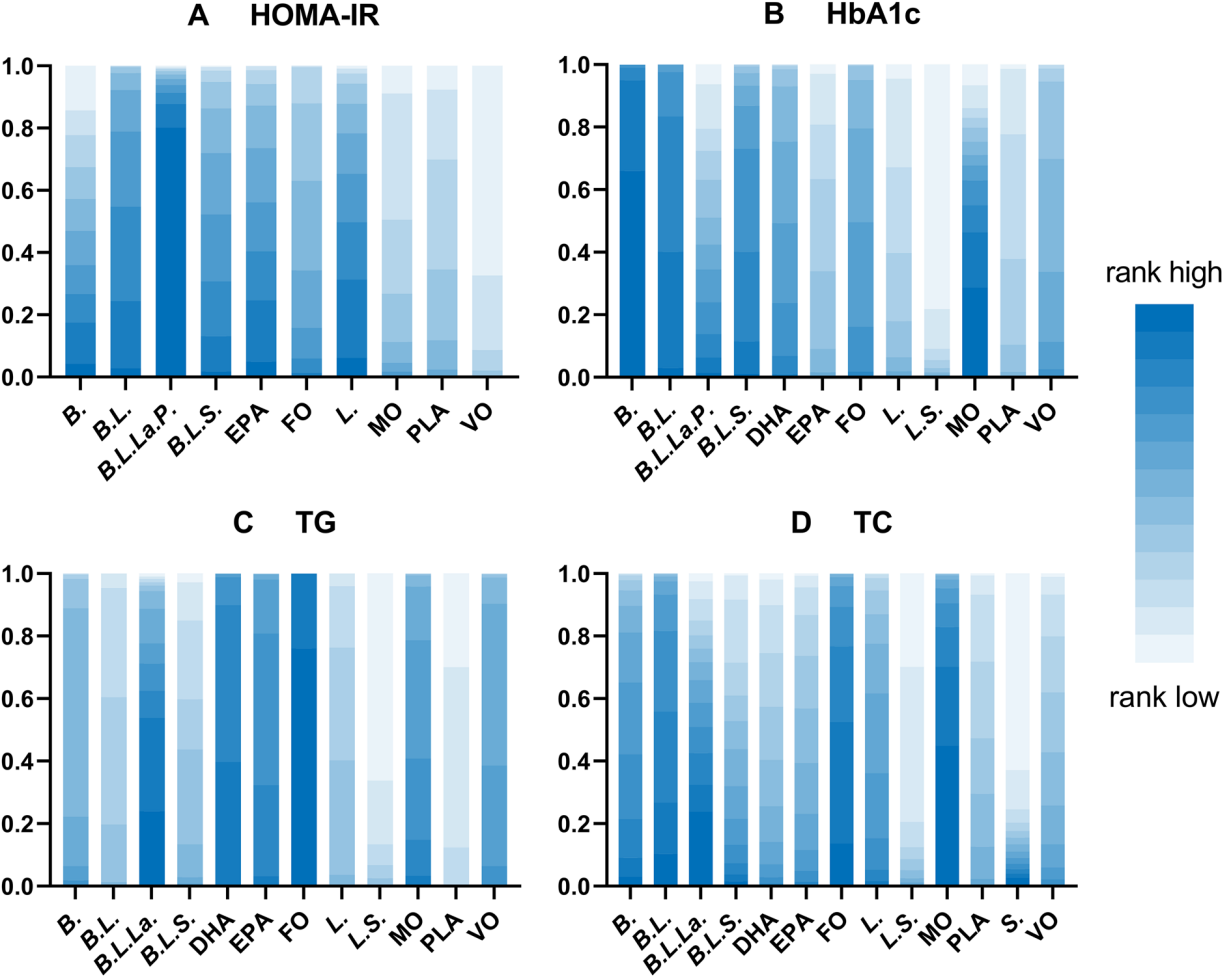
SUCRA analysis column chart. *FO* Fish oil, *MO* Mineral oil including paraffin oil, *VO* Vegetale oil including corn oil, olive oil, sunflower oil, flaxseed oil and perilla oil, *B. Bifidobacterium, L. Lactobacillus, La. Lactococcus, S. Streptococcus, P. Propionibacterium*, *PLA* Placebo

A total of 20 RCTs investigated the effect of fish oil and probiotics on HOMA-IR. The network diagram is shown in Fig. 3A. Due to low heterogeneity, the fixed effect model was used (I^2^ = 24%). According to SURCA analysis (ranking Additional file 1: Table S2 and column chart Fig. 4A) and League Table 3A), the combination of *Bifidobacterium, Lactobacillus, Lactococcus, and Propionibacterium* was the most effective in reducing HOMA-IR (SURCA = 0.935), followed by the combination of *Bifidobacterium* and *Lactobacillus* (Surca = 0.722) and *Lactobacillus* alone (Surca = 0.675). Additionally, all types of probiotics were more effective than fish oil in lowering HOMA-IR. Furthermore, 41 RCTs explored the effects of fish oil and probiotics on HbA1C. The network diagram is shown in Fig. 3B. Due to low heterogeneity (I^2^ = 45%), the fixed effect model was adopted. According to SUCRA analysis (ranking Additional file 1: Table S2 and column chart Fig. 4B) and league table (Table 3B), *Bifidobacterium* (Surca = 0.963) was the most effective in reducing HbA1c, followed by the combination of *Bifidobacterium* and *Lactobacillus* (Surca = 0.840) and the combination of *Bifidobacterium, Lactobacillus,* and *Streptococcus* (Surca = 0.729).

**Table 3:**
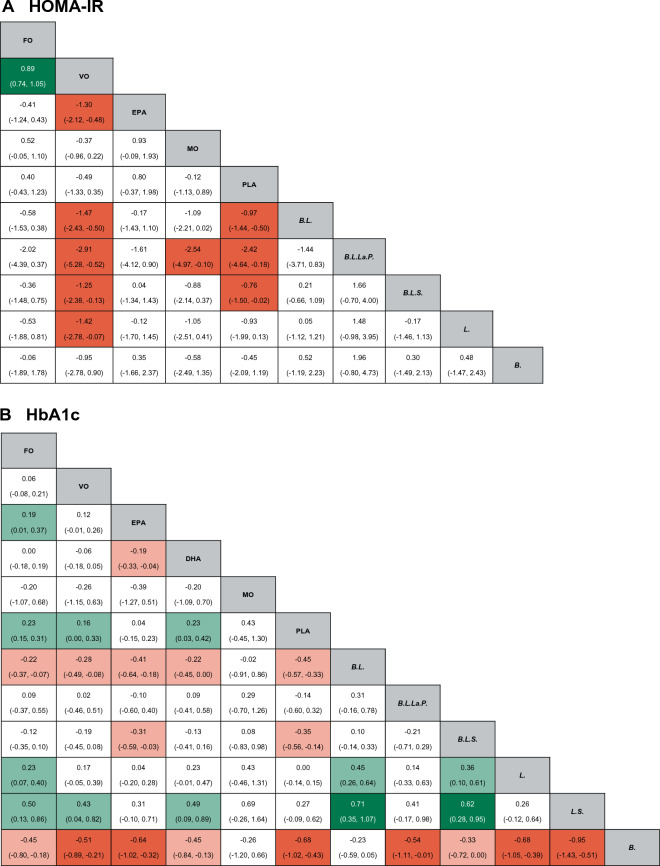

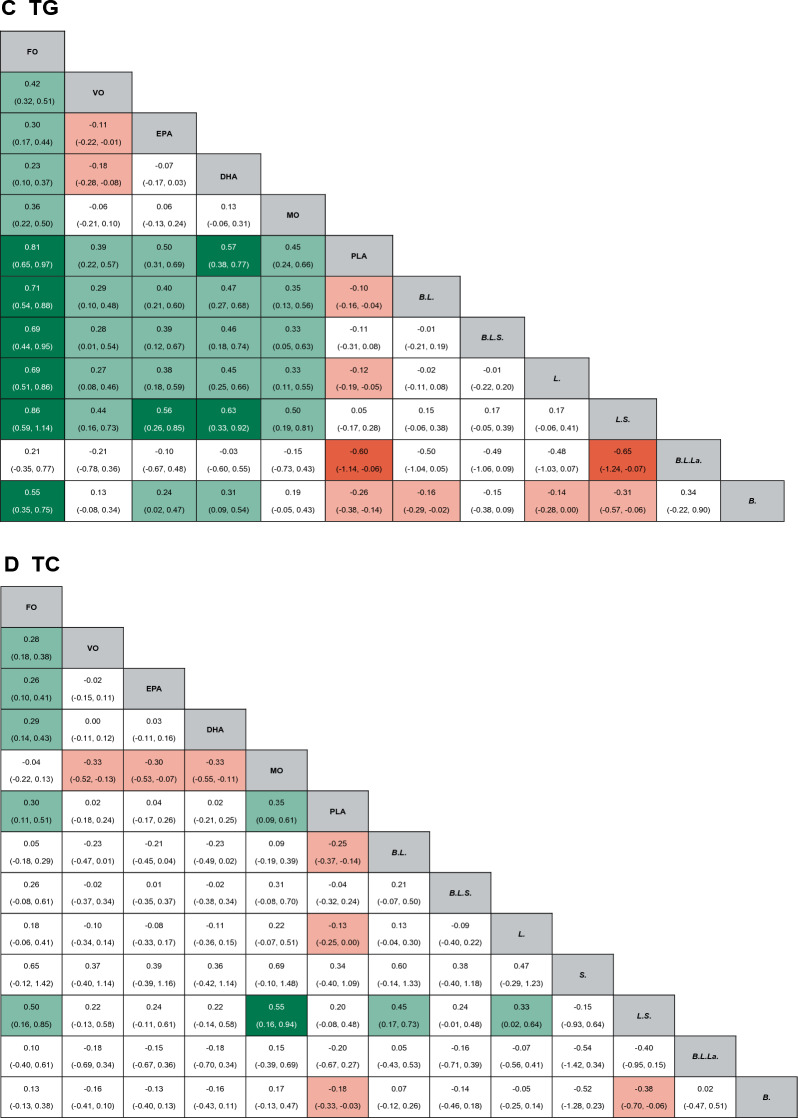
The league table of mean difference and 95% confidence intervals for primary outcome measures

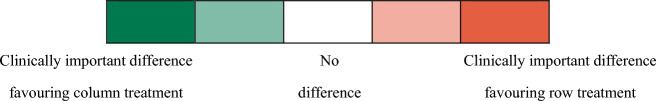

*FO* Fish oil, *MO* Mineral oil including paraffin oil, *VO* Vegetale oil including corn oil, olive oil, sunflower oil, flaxseed oil and perilla oil, *B. Bifidobacterium*, *L. Lactobacillus*, *La. Lactococcus*, *S. Streptococcus*, *P. Propionibacterium*, *PLA* Placebo

### Lipid metabolism

A total of 41 RCTs reported TG. The network diagram is shown in Fig. 3C. Owing to low heterogeneity (I^2^ = 37%), the fixed effect model was employed. According to SUCRA analysis (ranking Additional file 1: Table S2, column chart Fig. 4C) and league Table 3C, fish oil was the most effective in reducing TG (Surca = 0.978), followed by DHA (Surca = 0.844) and the combination of *Bifidobacterium, Lactobacillus,* and *Lactococcus* (Surca = 0.783). Furthermore, 43 RCTs analyzed the effects of fish oil and probiotics on TC. The network diagram is shown in Fig. 3D. Due to low heterogeneity (I^2^ = 30%), the fixed effect model was adopted. According to SUCRA results (ranking Additional file 1: Table S2 and column chart Fig. 4D) and League Table 3D), mineral oil (Surca = 0.902) had the best efficacy in reducing TC, followed by fish oil (Surca = 0.857) and the combination of *Bifidobacterium* and *Lactobacillus* (Surca = 0.803).

### Inflammatory markers

Seven RCTs reported inflammatory markers TNF-α, as shown in Additional file 1: Figure S1A. With low heterogeneity (I^2^ = 33%), the fixed effect model was adopted. According to SUCRA analysis (ranking Additional file 1: Table S2 and column chart Additional file 1: Fig. S2A) and League Additional file 1: Table S3A), fish oil (Surca = 0.839) was the most effective in reducing TNF-α, followed by mineral oil (Surca = 0.611) and *Lactobacillus* (Surca = 0.495).

### Adipocytokine

A total of 3 RCTs investigated the effects of fish oil and probiotics on leptin. The network diagram is shown in Additional file 1: Fig. S1B. With low heterogeneity (I^2^ = 16%), the fixed effect model was adopted. According to SUCRA analysis (ranking Additional file 1: Table S2 and column chart Fig. S2B) and League Additional file 1: Table S3B, fish oil (Surca = 0.712) was the most effective in reducing leptin levels, followed by mineral oil (Surca = 0.514) and *Lactobacillus* (Surca = 0.401). Moreover, a total of 8 RCTs explored the effects of fish oil and probiotics on adiponectin. The network diagram is shown in Additional file 1: Fig. S1C. With no heterogeneity (I^2^ = 0%), the fixed-effect model was adopted. According to SUCRA analysis (ranking Additional file 1: Table S2 and column chart Additional file 1: Fig. S2C) and League Additional file 1: Table S3C, fish oil (Surca = 0.742) was the most effective in increasing adiponectin levels, followed by *Lactobacillus* (Surca = 0.566) and vegetable oil (Surca = 0.469).

### Consistency check

**Fig. 5:**
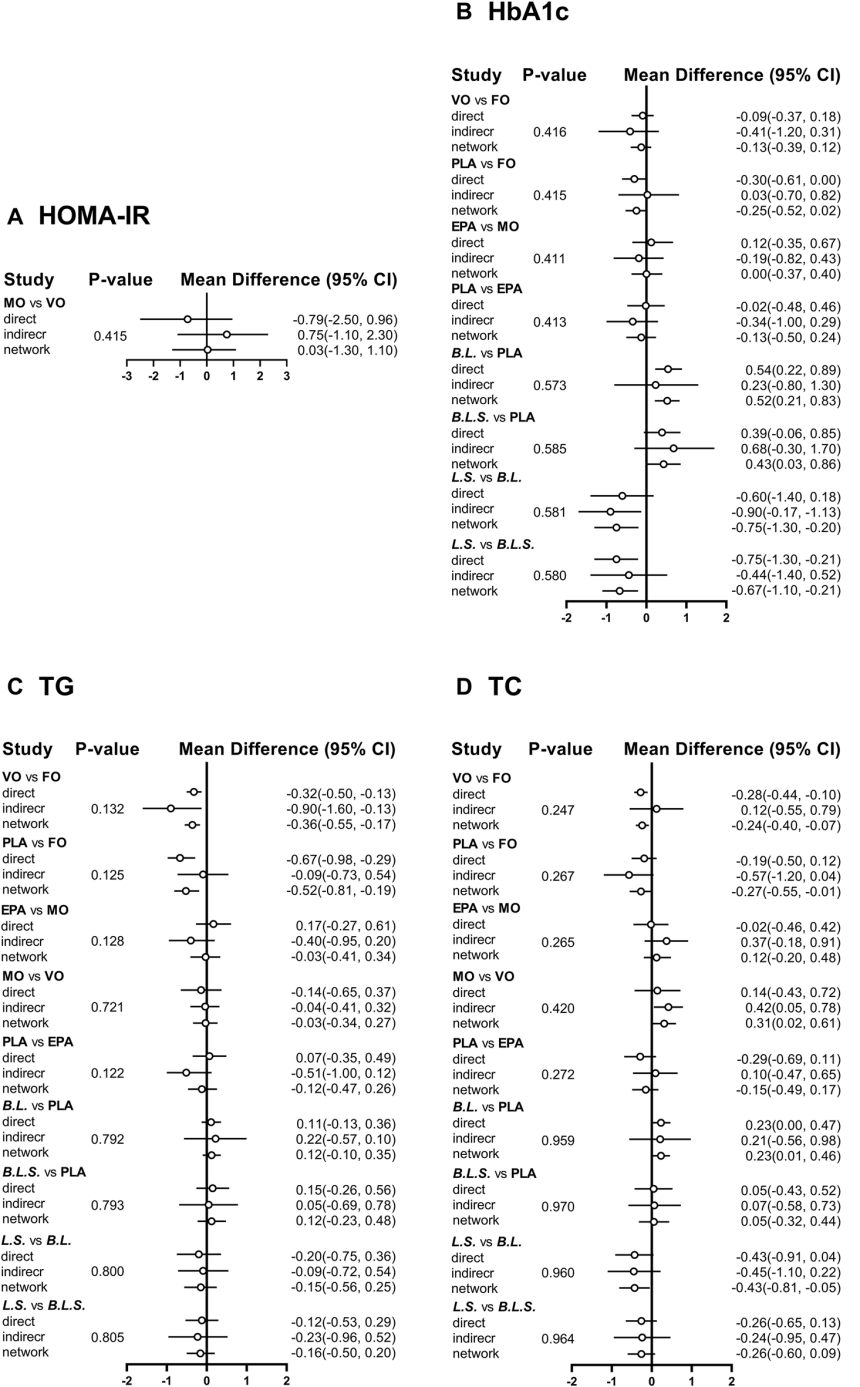
Node-splitting analysis diagram. *CI* Confidence intervals, *vs* Versus, *FO* Fish oil, *MO* Mineral oil including paraffin oil, *VO* Vegetale oil including corn oil, olive oil, sunflower oil, flaxseed oil and perilla oil, *B. Bifidobacterium; L., Lactobacillus; S., Streptococcus;* PLA, Placebo

Due to the absence of a network closed-loop structure for all secondary outcome measures, node-splitting analysis was employed only for the primary outcome measure. The analysis was conducted on selected interventions, including fish oil, EPA, mineral oil, vegetable oil, placebo, the combination of *Bifidobacterium* and *Lactobacillus*, the combination of *Lactobacillus* and *Streptococcus*, the combination of *Bifidobacterium, Lactobacillus,* and *Streptococcus*. The analysis showed that the P-values for direct, indirect, and network comparisons were greater than 0.05 (Fig. 5).

### Subgroup analysis

Subgroup analysis was conducted based on the intervention duration. Specifically, the intervention duration was < 12 weeks in 31 studies and ≥ 12 weeks in 29 studies. The heterogeneity analysis found that I^2^ in all studies was ≤ 50%, so a fixed effect model was adopted. In the group with an intervention duration of < 12 weeks, the combination of *Bifidobacterium*, *Lactobacillus*, *Lactococcus*, and *Propionibacterium* was the most effective in reducing HOMA-IR (Surca = 0.892). Furthermore, *Lactobacillus* demonstrated the highest efficacy in lowering HbA1c (Surca = 0.907), followed by the combination of *Bifidobacterium, Lactobacillus,* and *Streptococcus* (Surca = 0.734), and the combination of *Bifidobacterium* and *Lactobacillus* (Surca = 0.654). Fish oil was the most effective in reducing TG (Surca = 0.957). Vegetable oil was the most effective in reducing TC (Surca = 0.729), followed by mineral oil (Surca = 0.712) and EPA(Surca = 0.708). In the subgroup with intervention duration ≥ 12 weeks, the combination of *Bifidobacterium* and *Lactobacillus* demonstrated the highest efficacy in decreasing HOMA-IR (Surca = 0.910). *Bifidobacterium* exhibited the most effective in reducing HbA1c (Surca = 0.970). The combination of *Bifidobacterium, Lactobacillus* and *Lactococcus* (Surca = 0.945) was the most effective in reducing TG, followed by *Bifidobacterium* (Surca = 0.790) and fish oil (Surca = 0.627). Fish oil had the best effect on TC reduction (Surca = 0.799).

## Discussion

This study is the first to compare the efficacy of fish oil and probiotic supplementation on glucose and lipid metabolism in T2DM patients. Overall, the results demonstrated that fish oil (in the form of omega-3 fatty acids) was superior to EPA and DHA alone. Furthermore, fish oil significantly reduced both TG and TC levels in T2DM patients. Moreover, probiotics significantly ameliorated insulin resistance compared with fish oil. In addition, *Bifidobacterium* had a better effect on reducing HbA1c than other probiotic supplements and fish oil.

Regarding the glucose metabolism in T2DM patients, our NMA revealed that probiotics significantly reduced HOMA-IR levels compared to fish oil. Probiotics may improve insulin sensitivity by different mechanisms. First, probiotics are able to regulate the composition and function of gut microbiota [76]. For instance, probiotic-fermented blueberry juice improves insulin resistance in mice with a high-fat diet by regulating gut microbiota [77]. Another probiotic supplement, *Lactobacillus casei,* plays an antidiabetic role by reshaping the intestinal flora in T2DM rats [78]. Second, probiotics can ameliorate inflammation by secreting anti-inflammatory factors to reduce pro-inflammatory cytokines and lipopolysaccharide (LPS) levels, thereby improving insulin resistance and preserving the integrity of the intestinal epithelial cell wall. Proinflammatory cytokines induce the phosphorylation of insulin receptor substrate-1 serine and impede the insulin signaling pathway [79, 80]. LPS, as a component of the outer membrane of gram-negative bacteria, binds to the Toll-like receptor 4 (cluster of differentiation 14) to trigger the production of proinflammatory cytokines [81]. Third, probiotics can produce short-chain fatty acids (SCFAs), such as acetic acid, propionic acid, and butyric acid, through fermentation of dietary fiber. In individuals with T2DM, acetic acid can stimulate insulin secretion [82], while propionic and butyric acids inhibit the production of proinflammatory cytokines [83]. SCFAs can bind to the G protein-coupled receptor [84] and stimulate the production of downstream glucagon-like peptide-1 (GLP-1) and peptide yy [85], both of which improve insulin resistance. Fourth, probiotics can synthesize antioxidants to reduce oxidative stress, thus improving insulin sensitivity. Antioxidants can inhibit chain reactions by scavenging free radical intermediates and neutralizing free radicals [86]. Probiotics can significantly increase serum antioxidant indexes, such as glutathione, and reduce the expression of malondialdehyde in patients with diabetes [87]. Fifth, certain types of probiotics strengthen the mucus barrier by increasing the expression of mucin and stimulating mucus secretion. The intestinal barrier is crucial for preventing bacterial endotoxin from entering the blood and inducing inflammation and insulin resistance, which are important contributors to T2DM [88]. *Pediococcus acidilactici pA1c* increases the number of cupped cells, promotes the secretion of mucoglycoprotein, and maintains the appropriate length of intestinal villi [89]. *Bifidobacterium longum* and *Lactobacillus reuteri* can enhance mucus layer thickness [90]. *Lactobacillus spp.* can upregulate the expression of Mucin 2 and Mucin 3 [91] to enhance the intestinal mucosal barrier function.

Furthermore, our study found that T2DM patients who consumed *Bifidobacterium* had lower HbA1c than those who consumed other probiotics or fish oil. The mechanism by which *Bifidobacterium* lowers blood glucose may be similar to the mechanism just mentioned. It has been reported that *Bifidobacterium* can also decompose dietary fiber and produce metabolites such as SCFAs [92]. Meanwhile, *Bifidobacterium* can also indirectly increase the level of GLP-1 secreted by intestinal L cells by increasing the level of SCFAs [93]. Moreover, *Bifidobacterium* regulates the immune system and reduces chronic low-level inflammatory response, which can reduce blood glucose levels [94]. This finding may provide insights into the hypoglycemic mechanism of *Bifidobacterium* and the development of target drugs. Although it has been shown that fish oil can affect glucose metabolism, the role of omega-3 fatty acids in regulating blood glucose remains debatable [95]. Our NMA also found that fish oil was less effective than probiotics in regulating glucose metabolism in patients with T2DM.

Regarding lipid metabolism, fish oil was more effective in reducing TG and TC levels in T2DM patients than all probiotics. Similar results have also been documented in several meta-analyses [96–98]. Fish oil regulates TG levels through four possible mechanisms. First, omega-3 fatty acids can inhibit the expression of sterol regulatory element binding protein-1C in the liver. Consequently, this leads to a decrease in fatty acid synthase, resulting in reduced fatty acids in the liver. Ultimately, these mechanisms contribute to a reduction in triglyceride levels [99]. Second, omega-3 fatty acids promote fatty acid oxidation by increasing the metabolic rate of fatty acids to produce energy [100]. Third, omega-3 fatty acids reduce triglyceride synthesis by inhibiting phosphatidic acid phosphatase and diacylglycerol acyltransferase [101]. Fourth, omega-3 fatty acids can increase the expression of lipoprotein lipase (LPL). LPL is a key enzyme involved in the removal of triglycerides from circulating triglyceride-rich lipoproteins such as very low density lipoprotein and chylomicron. Increased LPL expression promotes the conversion and clearance of triglycerides, thereby reducing their levels in the blood [102]. These regulatory effects can affect the synthesis, oxidation and clearance of triglycerides. However, the exact mechanisms are still being studied and may be influenced by individual differences.

Despite the role of fish oil in reducing total cholesterol levels, the mechanism of the relationship between fish oil and cholesterol remains to be elucidated. Previous meta-analyses have found that omega-3 fatty acids in fish oil could elevate the concentration of high density lipoprotein cholesterol (HDL-c) in blood [96, 97]. HDL-c facilitates the transportation of cholesterol in the blood and tissues back to the liver for metabolism and excretion. A previous study showed that EPA could lower low density lipoprotein cholesterol (LDL-c) concentrations in blood [98]. Interestingly, based on our SUCRA results, paraffin oil, as a mineral oil, was the most effective intervention for reducing TC levels. However, long-term oral administration of mineral oil can lead to increased intestinal permeability, possibly have proinflammatory effects, and cause reduced TC levels. It may raise concerns about the use of mineral oil as a placebo in clinical studies [103]. The number of available studies on this topic is limited, and further research on the effectiveness of fish oil in reducing cholesterol levels is needed.

In terms of the inflammatory response, our results revealed that fish oil was more effective than probiotics in reducing TNF-α in T2DM patients. TNF-α, a proinflammatory cytokine, is primarily secreted by macrophages and monocytes and is involved in inflammatory and immune responses. Several studies showed that fish oil could not only reduce TNF-α but also inhibit nuclear factor-κB activation, one of the major inflammatory transcription factors [104, 105]. In vitro and in vivo studies demonstrated that EPA and DHA could inhibit the production of TNF-α [106]. Furthermore, our study also found that fish oil supplementation also reduced leptin concentration in blood and increased adiponectin levels. This result is consistent with a previous meta-analysis, which indicates that omega-3 fatty acids reduce leptin levels and increase adiponectin levels in T2DM patients [107]. Leptin is an adipocyte-derived hormone that regulates appetite and energy metabolism to control body weight and energy balance [108]. An increased leptin concentration in blood is associated with insulin resistance and obesity in T2DM individuals [109]. However, the effect of fish oil on leptin levels remains debatable [110, 111]. Adiponectin is a hormone secreted by fat cells and it can promote glucose utilization, inhibit fatty acid oxidation and inflammation, and thus increase insulin sensitivity [112]. Adiponectin synthesis begins when the omega-3 fatty acids in fish oil bind to peroxisome proliferator-activated receptor-γ (PPAR-γ). When PPAR-γ exerts an antagonistic effect, the effect of omega-3 fatty acids on adiponectin is blocked [113]. Alternatively, omega-3 fatty acids inhibit transient receptor potentials in mature adipocytes to regulate calcium channels and thus, enhance adiponectin production [114]. It is important to note that the results should be cautiously interpreted due to the limited number of the included RCTs.

Node-splitting analysis for consistency showed P > 0.05 in direct, indirect, and network comparison of various interventions, suggesting that the included studies had good consistency. No heterogeneity was found in the network analysis (both I^2^ ≤ 50%), and thus sensitivity analysis was not performed. In the subgroup analysis, the results in the subgroup of intervention duration ≥ 12 weeks were different from the overall analysis results in TG reduction. The SURCA analysis showed that fish oil was more effective than probiotics in regulating lipid metabolism in T2DM patients, and probiotics were superior to fish oil in regulating glucose metabolism. In the subgroup with intervention duration ≥ 12 weeks, the combination of *Bifidobacterium, Lactobacillus,* and *Lactococcus* was the most effective in reducing TG, followed by *Bifidobacterium* and fish oil. However, it is important to note that in this subgroup, the combination of *Bifidobacterium, Lactobacillus* and *Lactococcus* was only used in the study by Sabico [58], and *Bifidobacterium* was only reported in the study by Chaiyasut [73]. Since the two interventions was only used in one study, respectively, our results may be biased due to limited data, and publication bias may exist. It may also indicate that fish oil was more effective in reducing TC only at the early stage (< 12 weeks). Therefore, high-quality RCTs with a large and diverse population are required to validate these findings.

Our findings provide crucial insights into the clinical effects of fish oil and probiotics on T2DM. Fish oil is found to outperform probiotics in reducing triglycerides and total cholesterol levels, thereby mitigating the risk of cardiovascular disease in T2DM. Proper supplementation of fish oil may improve cardiovascular conditions among these patients. Furthermore, probiotics, especially those containing strains like bifidobacteria, are effective in lowering blood sugar levels compared to fish oil. This could contribute to the control of blood sugar and the overall management of diabetes. These findings may assist clinicians in designing more effective and personalized treatment plans. This study not only introduces new insights into the management of T2DM but also provides a deeper understanding of this disease.

There are several limitations in this study. Firstly, other inflammatory markers, such as interleukin-6, procalcitonin, and erythrocyte sedimentation rate, are more widely used than TNF-α in clinical practice. Unfortunately, due to limited data, not all interventions were linked in a network, so further analysis could not be conducted. Secondly, the inconsistency of fish oil and probiotic dosages across studies may lead to biased results. Finally, adherence to interventions is important, especially for those implemented outside hospitals. However, the description of patient compliance in the included RCTs was relatively limited, which may affect the full assessment of the treatment effects. Although we tried to take this into account in the study design, data on patient compliance remain limited. Future studies should consider the potential impact of patient compliance on outcomes.

## Conclusions

Regarding lipid metabolism in T2DM patients, fish oil significantly reduced TG and TC levels compared to probiotics. Probiotics were more effective than fish oil in improving insulin resistance. Particularly, *Bifidobacterium* was more effective in reducing blood glucose levels than other probiotic supplements and fish oil. Nevertheless, high-quality and large-scale RCTs are required to validate these results. Further research should also explore the specific mechanisms and optimal treatment options of probiotics and fish oil to improve glucose and lipid metabolism in T2DM patients.

### Supplementary Information

**Additional file 1: Table S1.** Complete list of 3 electronic library search terms. **Table S2.** SUCRA ranking table for each outcome indicator. **Table S3.** The league table of mean difference and 95% confidence intervals for secondary outcome indicators. **Fig. S1**. The network plots. **Fig. S2**. SUCRA analysis column chart.

## Data Availability

The datasets generated during and/or analysed during the current study are available from the corresponding authors on reasonable request.
