# Supplementary material for: Comparison of the efficacy of fish oil and probiotic supplementation on glucose and lipid metabolism in patients with type 2 diabetes: a systematic review and network meta-analysis
Source: Diabetol Metab Syndr. 2024 Jan 22;16:25. doi: 10.1186/s13098-024-01266-3 (PMC10804729; doi:10.1186/s13098-024-01266-3)
Supplement: Supplementary file 1 — Additional file 1: Table S1. Complete list of 3 electronic library search terms. Table S2. SUCRA ranking table for each outcome indicator. Table S3. The league table of mean difference and 95% confidence intervals for secondary outcome indicators. Fig. S1. The network plots. Fig. S2. SUCRA analysis column chart. [file 13098_2024_1266_MOESM1_ESM.docx]

| **Pubmed(n=146)** |  | **Embase(n=237)** |  | **Cochrane Library(n=445)** |
| --- | --- | --- | --- | --- |
| **#1** "adult onset diabetes"[Title/Abstract] OR "adult onset diabetes mellitus"[Title/Abstract] OR "DM 2"[Title/Abstract] OR "insulin independent diabetes"[Title/Abstract] OR "insulin independent diabetes mellitus"[Title/Abstract] OR "ketosis resistant diabetes mellitus"[Title/Abstract] OR "maturity onset diabetes"[Title/Abstract] OR "maturity onset diabetes mellitus"[Title/Abstract] OR "maturity onset diabetes of the young"[Title/Abstract] OR "MODY"[Title/Abstract] OR "NIDDM"[Title/Abstract] OR "non insulin dependent diabetes"[Title/Abstract] OR "non insulin dependent diabetes mellitus"[Title/Abstract] OR "noninsulin dependent diabetes"[Title/Abstract] OR "noninsulin dependent diabetes mellitus"[Title/Abstract] OR "Slow Onset Diabetes Mellitus"[Title/Abstract] OR "Stable Diabetes Mellitus"[Title/Abstract] OR "T2DM"[Title/Abstract] OR "type 2 diabetes"[Title/Abstract] OR "type 2 diabetes mellitus"[Title/Abstract] OR "type II diabetes"[Title/Abstract] OR "type II diabetes mellitus"[Title/Abstract] **Results:** 197,900  **#2** type 2 diabetes mellitus[MeSH Terms] **Results:** 175,072  **#3** "ameu"[Title/Abstract] OR "efamed"[Title/Abstract] OR "epax"[Title/Abstract] OR "epax 5000"[Title/Abstract] OR "feniko"[Title/Abstract] OR "Fish Liver Oils"[Title/Abstract] OR "Fish Oil"[Title/Abstract] OR "Fish Oils"[Title/Abstract] OR "himega"[Title/Abstract] OR "k 85"[Title/Abstract] OR "k 85 fish oil preparation"[Title/Abstract] OR "lachs 550"[Title/Abstract] OR "lipitac"[Title/Abstract] OR "maxepa"[Title/Abstract] OR "olemar"[Title/Abstract] OR "omegaven"[Title/Abstract] OR "optimepa"[Title/Abstract] OR "pikasol"[Title/Abstract] OR "promega"[Title/Abstract] OR "super epa"[Title/Abstract] OR "superepa"[Title/Abstract] OR "tuna oil"[Title/Abstract] **Results:** 13,046  **#4** Fish Oils[MeSH Terms] **Results:** 32,990  **#5** "probiotic agent"[Title/Abstract] OR "Probiotics"[Title/Abstract] OR "Probiotic"[Title/Abstract] **Results:** 40,792  **#6** Probiotics[MeSH Terms] **Results:** 25,097  **#7** "randomised controlled study "[Title/Abstract] OR "randomised controlled trial "[Title/Abstract] OR "randomized controlled study "[Title/Abstract] OR "randomized controlled trial"[Title/Abstract] **Results:** 160,104  **#8** Randomized Controlled Trial[MeSH Terms] **Results:** 169,338  **#9** (#1 OR #2) AND ((#3 OR #4) OR (#5 OR #6)) AND (#7 OR #8) **Results:** 146 |  | **#1** 'adult onset diabetes':ti,ab,kw OR 'adult onset diabetes mellitus':ti,ab,kw OR 'dm 2':ti,ab,kw OR 'insulin independent diabetes':ti,ab,kw OR 'insulin independent diabetes mellitus':ti,ab,kw OR 'ketosis resistant diabetes mellitus':ti,ab,kw OR 'maturity onset diabetes':ti,ab,kw OR 'maturity onset diabetes mellitus':ti,ab,kw OR 'maturity onset diabetes of the young':ti,ab,kw OR 'mody':ti,ab,kw OR 'niddm':ti,ab,kw OR 'non insulin dependent diabetes':ti,ab,kw OR 'non insulin dependent diabetes mellitus':ti,ab,kw OR 'noninsulin dependent diabetes':ti,ab,kw OR 'noninsulin dependent diabetes mellitus':ti,ab,kw OR 'slow onset diabetes mellitus':ti,ab,kw OR 'stable diabetes mellitus':ti,ab,kw OR 't2dm':ti,ab,kw OR 'type 2 diabetes':ti,ab,kw OR 'type 2 diabetes mellitus':ti,ab,kw OR 'type ii diabetes':ti,ab,kw OR 'type ii diabetes mellitus':ti,ab,kw **Results:** 305,227  **#2** 'non insulin dependent diabetes mellitus'/exp **Results:** 347,902  **#3** 'ameu':ti,ab,kw OR 'efamed':ti,ab,kw OR 'epax':ti,ab,kw OR 'epax 5000':ti,ab,kw OR 'feniko':ti,ab,kw OR 'fish liver oils':ti,ab,kw OR 'fish oil':ti,ab,kw OR 'fish oils':ti,ab,kw OR 'himega':ti,ab,kw OR 'k 85':ti,ab,kw OR 'k 85 fish oil preparation':ti,ab,kw OR 'lachs 550':ti,ab,kw OR 'lipitac':ti,ab,kw OR 'maxepa':ti,ab,kw OR 'olemar':ti,ab,kw OR 'omegaven':ti,ab,kw OR 'optimepa':ti,ab,kw OR 'pikasol':ti,ab,kw OR 'promega':ti,ab,kw OR 'super epa':ti,ab,kw OR 'superepa':ti,ab,kw OR 'tuna oil':ti,ab,kw **Results:** 18,546  **#4** 'fish oil'/exp **Results:** 19,617  **#5** 'probiotic agent':ti,ab,kw OR 'probiotics':ti,ab,kw OR 'probiotic':ti,ab,kw **Results:** 50,203  **#6** 'probiotic agent'/exp **Results:** 54,665  **#7** 'randomised controlled study':ti,ab,kw OR 'randomised controlled trial':ti,ab,kw OR 'randomized controlled study':ti,ab,kw OR 'randomized controlled trial':ti,ab,kw **Results:** 211,162  **#8** 'randomized controlled trial'/exp **Results:** 800,625  **#9** (#1 OR #2) AND (#3 OR #4 OR #5 OR #6) AND (#7 OR #8) **Results:** 237 |  | **#1** ('adult onset diabetes' OR 'adult onset diabetes mellitus' OR 'DM 2' OR 'insulin independent diabetes' OR 'insulin independent diabetes mellitus' OR 'ketosis resistant diabetes mellitus' OR 'maturity onset diabetes' OR 'maturity onset diabetes mellitus' OR 'maturity onset diabetes of the young' OR 'MODY' OR 'NIDDM' OR 'non insulin dependent diabetes' OR 'non insulin dependent diabetes mellitus' OR 'noninsulin dependent diabetes' OR 'noninsulin dependent diabetes mellitus' OR 'Slow Onset Diabetes Mellitus' OR 'Stable Diabetes Mellitus' OR 'T2DM' OR 'type 2 diabetes' OR 'type 2 diabetes mellitus' OR 'type II diabetes' OR 'type II diabetes mellitus'):ti,ab,kw **Results:** 69,689  **#2** MeSH descriptor: [Diabetes Mellitus, Type 2] explode all trees **Results:** 23,476  **#3** ('ameu' OR 'efamed' OR 'epax' OR 'epax 5000' OR 'feniko' OR 'Fish Liver Oils' OR 'Fish Oil' OR 'Fish Oils' OR 'himega' OR 'k 85' OR 'k 85 fish oil preparation' OR 'lachs 550' OR 'lipitac' OR 'maxepa' OR 'olemar' OR 'omegaven' OR 'optimepa' OR 'pikasol' OR 'promega' OR 'super epa' OR 'superepa' OR 'tuna oil'):ti,ab,kw **Results:** 5,447  **#4** MeSH descriptor: [Fish Oils] explode all trees **Results:** 4,204  **#5** ('probiotic agent' OR 'Probiotics' OR 'Probiotic'):ti,ab,kw **Results:** 9,774  **#6** MeSH descriptor: [Probiotics] explode all trees **Results:** 3,072  **#7** ('randomised controlled study ' OR 'randomised controlled trial ' OR 'randomized controlled study ' OR 'randomized controlled trial'):ti,ab,kw **Results:** 807,733  **#8** MeSH descriptor: [Randomized Controlled Trial] explode all trees **Results:** 25,729  **#9** (#1 OR #2) AND ((#3 OR #4) OR (#5 OR #6)) AND (#7 OR #8) **Results:** 445 |

**Table S1** Complete list of 3 electronic library search terms (Updated December 13, 2023)

**Table S2** SUCRA ranking table for each outcome indicator

| **HOMA-IR** | *B.L.La.P.* | *B.L.* | *L.* | EPA | *B.L.S.* | *B.* | FO | PLA | MO | VO |  |  |  |
| --- | --- | --- | --- | --- | --- | --- | --- | --- | --- | --- | --- | --- | --- |
|  | 0.935 | 0.722 | 0.675 | 0.645 | 0.614 | 0.466 | 0.454 | 0.234 | 0.207 | 0.049 |  |  |  |
| **HbA1c** | *B.* | *B.L.* | *B.L.S.* | MO | DHA | FO | VO | *B.L.La.P.* | EPA | *L.* | PLA | *L.S.* |  |
|  | 0.963 | 0.840 | 0.729 | 0.681 | 0.588 | 0.583 | 0.464 | 0.438 | 0.260 | 0.208 | 0.206 | 0.038 |  |
| **TG** | FO | DHA | *B.L.La.* | EPA | MO | VO | *B.* | *L.* | *B.L.S.* | *B.L.* | PLA | *L.S.* |  |
|  | 0.978 | 0.844 | 0.783 | 0.740 | 0.668 | 0.576 | 0.469 | 0.288 | 0.274 | 0.252 | 0.075 | 0.052 |  |
| **TC** | MO | FO | *B.L.* | *B.* | *B.L.La.* | *L.* | EPA | *B.L.S.* | VO | DHA | PLA | *S.* | *L.S.* |
|  | 0.902 | 0.857 | 0.803 | 0.666 | 0.645 | 0.564 | 0.413 | 0.412 | 0.357 | 0.346 | 0.296 | 0.141 | 0.099 |
| **TNF-α** | FO | MO | *L.* | PLA | VO |  |  |  |  |  |  |  |  |
|  | 0.839 | 0.611 | 0.495 | 0.487 | 0.068 |  |  |  |  |  |  |  |  |
| **Leptin** | FO | MO | *L.* | PLA |  |  |  |  |  |  |  |  |  |
|  | 0.712 | 0.514 | 0.401 | 0.373 |  |  |  |  |  |  |  |  |  |
| **Adiponectin** | FO | *L.* | VO | PLA | MO |  |  |  |  |  |  |  |  |
|  | 0.742 | 0.566 | 0.469 | 0.458 | 0.266 |  |  |  |  |  |  |  |  |

FO, Fish oil; MO, Mineral oil including paraffin oil; VO, Vegetale oil including corn oil, olive oil, sunflower oil, flaxseed oil and perilla oil; *B., Bifidobacterium; L., Lactobacillus; La., Lactococcus; S., Streptococcus; P., Propionibacterium*; PLA, Placebo

**Table S3** The league table of mean difference and 95% confidence intervals for secondary outcome indicators

**A TNF-α**

| **FO** |  |  |  |  |
| --- | --- | --- | --- | --- |
| 5.05  (1.38, 8.72) | **VO** |  |  |  |
| -0.51  (-15.19, 14.13) | -5.55  (-20.65, 9.53) | **MO** |  |  |
| 0.90  (-0.21, 2.00) | -4.16  (-8.03, -0.31) | 1.41  (-13.28, 16.14) | **PLA** |  |
| 0.89  (-0.23, 2.02) | -4.16  (-8.03, -0.32) | 1.40  (-13.28, 16.13) | 0.00  (-0.20, 0.19) | ***L.*** |

**B Leptin**

| **FO** |  |  |  |
| --- | --- | --- | --- |
| 1.50  (-9.94, 12.99) | **MO** |  |  |
| 2.82  (-4.24, 9.93) | 1.31  (-12.13, 14.76) | **PLA** |  |
| 2.72  (-5.06, 10.51) | 1.21  (-12.72, 15.01) | -0.10  (-3.36, 3.12) | ***L.*** |

**C Adiponectin**

| **FO** |  |  |  |  |
| --- | --- | --- | --- | --- |
| -0.16  (-0.28, -0.04) | **VO** |  |  |  |
| -0.73  (-1.74, 0.28) | -0.57  (-1.58, 0.44) | **MO** |  |  |
| -0.33  (-5.88, 5.27) | -0.17  (-5.72, 5.44) | 0.39  (-5.24, 6.08) | **PLA** |  |
| -0.11  (-5.74, 5.57) | 0.06  (-5.59, 5.74) | 0.63  (-5.11, 6.39) | 0.22  (-0.80, 1.25) | ***L.*** |

|  |  |  |  |  |
| --- | --- | --- | --- | --- |

| Clinically important difference  favouring column treatment |  | No  difference |  | Clinically important difference  favouring row treatment |
| --- | --- | --- | --- | --- |

FO, Fish oil; MO, Mineral oil including Paraffin oil; VO, Vegetale oil including Corn oil, Olive oil, Sunflower oil, Flaxseed oil and Perilla oil; *L., Lactobacillus*; Placebo, PLA

**Fig. S1** The network plots


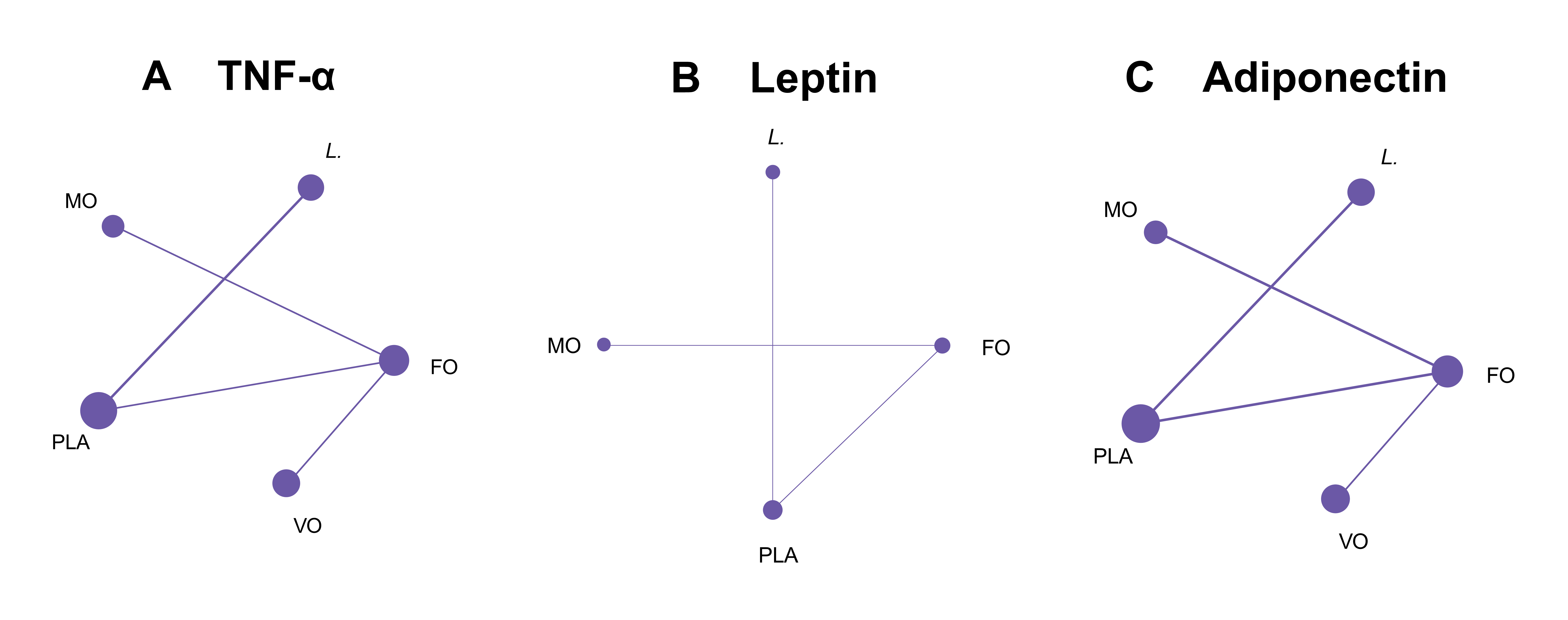


FO, Fish oil; MO, Mineral oil including Paraffin oil; VO, Vegetale oil including Corn oil, Olive oil, Sunflower oil, Flaxseed oil and Perilla oil; *L., Lactobacillus*; Placebo, PLA

**Fig. S2** SUCRA analysis column chart


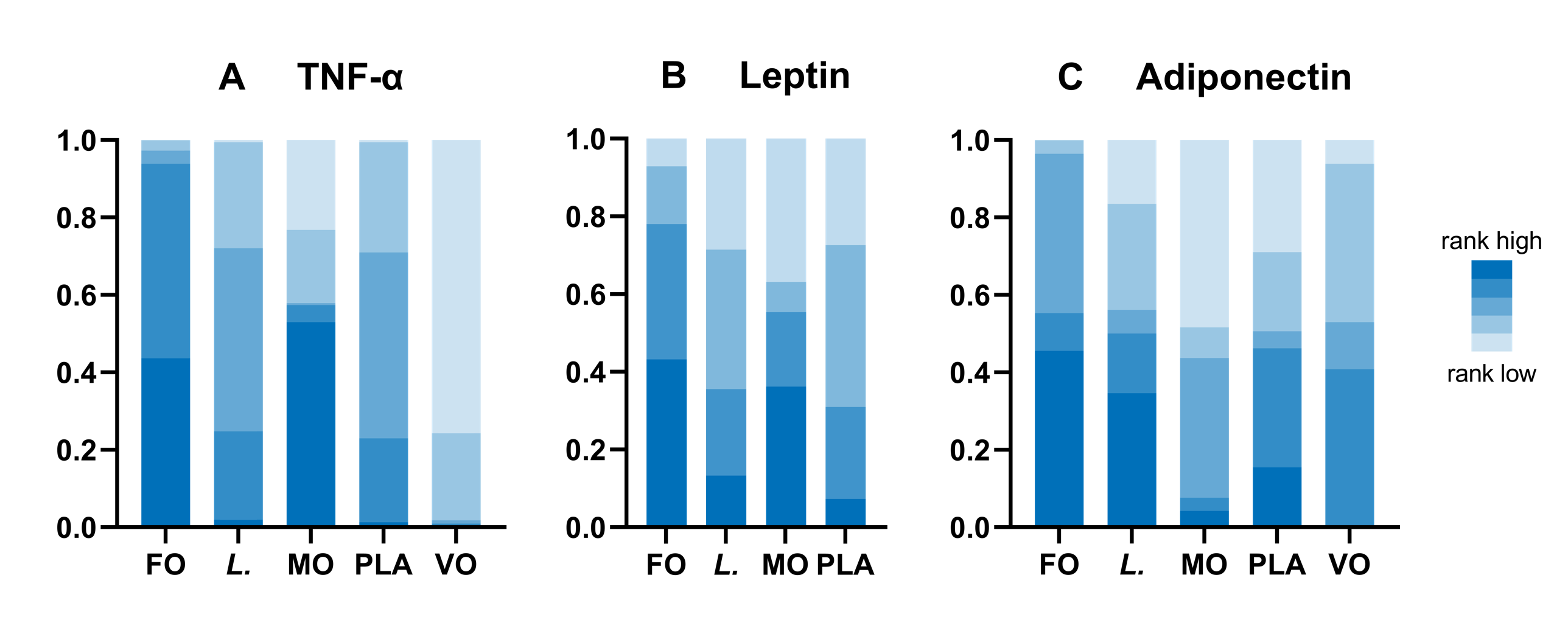


FO, Fish oil; MO, Mineral oil including Paraffin oil; VO, Vegetale oil including Corn oil, Olive oil, Sunflower oil, Flaxseed oil and Perilla oil; *L., Lactobacillus*; Placebo, PLA
